# The fourth COVID-19 vaccine dose increased the neutralizing antibody response against the SARS-CoV-2 Omicron (B.1.1.529) variant in a diverse Brazilian population

**DOI:** 10.1128/spectrum.02857-23

**Published:** 2023-11-01

**Authors:** Jéssica Pires Farias, Robert Andreata-Santos, Ruth Dalety da Silva Brito, Milena Silva Souza, Mayanna Moreira Costa Fogaça, Josilene Ramos Pinheiro, Edgar Ferreira da Cruz, Willian Liang, Rafael da Conceição Simões, Wilson Barros Luiz, Alexander Birbrair, Paloma Oliveira Vidal, Juliana Terzi Maricato, Carla Torres Braconi, Luís Carlos de Souza Ferreira, Luiz Mário Ramos Janini, Jaime Henrique Amorim

**Affiliations:** 1 Western Bahia Virology Institute, Center of Biological Sciences and Health, Federal University of Western Bahia, Barreiras, Bahia, Brazil; 2 Department of Microbiology, Immunology and Parasitology, Paulista School of Medicine, Federal University of São Paulo (UNIFESP), São Paulo, São Paulo, Brazil; 3 Department of Biological Sciences, State University of Santa Cruz, Ilhéus, Bahia, Brazil; 4 Department of Dermatology, School of Medicine and Public Health, University of Wisconsin-Madison, Madison, Wisconsin, USA; 5 Department of Pathology, Federal University of Minas Gerais, Belo Horizonte, Minas Gerais, Brazil; 6 Department of Radiology, Columbia University Medical Center, New York, New York, USA; 7 Department of Microbiology, Biomedical Sciences Institute, University of São Paulo, São Paulo, São Paulo, Brazil; 8 Scientific Platform Pasteur USP, University of São Paulo, São Paulo, São Paulo, Brazil; 9 Department of Medicine, Division of Infectology, Federal University of São Paulo, São Paulo, São Paulo, Brazil; Fred Hutchinson Cancer Research Center, Seattle, Washington, USA

**Keywords:** Omicron subvariants, fourth dose, vaccines, neutralization, epitopes

## Abstract

Most of the original immunization regimens against severe acute respiratory syndrome coronavirus 2 (SARS-CoV-2) were composed of two doses, followed by a subsequent third booster dose to control the Omicron variant and hence coronavirus disease 2019 (COVID-19). However, most data generated regarding the fourth dose were not based on the general population. Therefore, this study aimed to verify the effect of the fourth COVID-19 vaccine dose in a diverse Brazilian population. This retrospective observational study was conducted between May and September 2022. We gathered data on the vaccine regimens and COVID-19 serologic status from 266 healthy volunteers who received three or four vaccine doses, as well as COVID-19 diagnosis and viral genome sequencing from 457 patients with flu-like symptoms. In addition, we conducted immunoinformatic analysis to assess the conserved epitopes in the locally circulating viruses. We showed that the fourth dose did not increase the serum levels of antiviral antibodies, as measured by enzyme-linked immunosorbent assay. However, it significantly increased neutralizing antibody (NAb) titers against the Omicron variant. All viral sequences generated in this study were Omicron subvariants, mainly B.A.5.1. Notably, most NAb epitopes present in the wild-type SARS-CoV-2 were not detected among the circulating Omicron subvariants. None of the volunteers who received the third or fourth doses presented COVID-19 for at least 1 year before the study period. Altogether, these results indicate that the fourth vaccine dose increases the serum levels of NAbs that recognize highly conserved epitopes in Omicron subvariants.

**IMPORTANCE:**

Several additional COVID-19 vaccine doses were administered in the Brazilian population to prevent the disease caused by the B.1.1.529 (Omicron) variant. The efficacy of a third dose as a booster is already well described. However, it is important to clarify the humoral immune response gain induced by a fourth dose. In this study, we evaluate the effect of the fourth COVID-19 vaccine dose in a diverse Brazilian population, considering a real-life context. Our study reveals that the fourth dose of the COVID-19 vaccine increased the neutralizing antibody response against SARS-CoV-2 Omicron and significantly contributed in the reduction of the disease caused by this variant.

## INTRODUCTION

The emergence of the severe acute respiratory syndrome coronavirus 2 (SARS-CoV-2) caused the coronavirus disease 2019 (COVID-19) in 2019, more than 770 million people have been affected globally, which has resulted in more than 6.9 million deaths (1). Due to great efforts across the globe, several vaccine formulations were clinically validated in record time and used to fight the COVID-19 pandemic (2, 3). Vaccination is the most efficient strategy for controlling COVID-19, particularly in severe and lethal cases (4, 5). Despite this progress, mutations in the main vaccine target (the spike protein) have resulted in the evolution of SARS-CoV-2 variants that are capable of circumventing the neutralization activity of serum antibodies elicited in vaccinated or infected individuals (6 – 8). This has raised concerns with regard to vaccine efficacy, especially formulations based on wild-type SARS-CoV-2, which does not contain the mutated epitopes.

In Brazil, the most commonly used vaccine formulations are Sinovac-CoronaVac (based on a purified inactivated virus) (9), Oxford/Astrazeneca (AZD1222 or ChAdOx1-S), and Janssen (Ad26.COV2.S), which are based on an adenovirus vector encoding the spike protein of SARS-CoV-2 (10, 11), and Pfizer (BNT126b2, RNA-based vaccine) (12, 13). Except for the Janssen vaccine, the original immunization regimens were composed of two doses. We previously demonstrated that the antibody levels induced by two vaccine doses wane over time and are restored by a third booster dose (14). In addition, such a booster can induce neutralizing antibodies (NAbs) that protect individuals from infection by Omicron, a variant of concern (VOC), as well as its subvariants (15, 16). Thus, the importance of a third vaccine dose is widely recognized for combating the COVID-19 pandemic.

As an example of the efficacy of the vaccination policy in Brazil, the number of COVID-19 cases and deaths diminished over time, according to the progress of vaccine dose administration in the study area, Barreiras, Brazil (Table 1), based on official municipal data (17, 18). From the beginning of SARS-CoV-2 circulation in the study area in 2020, there was an increase in the number of cases and deaths in mid-2021 as a result of the replacement of viral strains in the early stages of the pandemic by the Gamma VOC (19). This situation was combated with completion of the original immunization regimen (two doses) in the city population and administration of additional doses.

**TABLE 1:** Official COVID-19 epidemiological and vaccination information of the city of Barreiras during the period of study.

Variable	2020^*a*^	2021^*b*^	2022^*c*^
Number of cases (year)^*d*^	8,085	12,356	6,691
Number of cases (May to September)^*e*^	4,676	7,171	2,865
ICU^*f*^ occupancy rate (May to September)	35%	53.60%	0%
Deaths (May to September)	82	144	2

Administered vaccine doses^*g*^	Vaccination coverage (%)		
First dose	-	82.28	100
Second dose	-	41.91	99.96
Third dose	-	0.23	50.78
Fourth dose	-	-	12.03

^*a*^ Variables computed in the year of 2020.

^*b*^ Variables computed in the year of 2021.

^*c*^ Variables computed in the year of 2022.

^*d*^ Events computed across the year.

^*e*^ Events computed in the period of May to September of each year.

^*f*^ ICU, intensive care unit.

^*g*^ Events computed until September of each year.

The fourth vaccine dose against COVID-19 has been studied previously (20 – 24). However, these studies were based on populations comprising elderly and immunocompromised individuals. Little is known about the effect of the fourth vaccine dose on the general population, which is comprised of individuals of different ages, in different occupations, and with varying health conditions. Therefore, in the present study, we aimed to evaluate the impact of the fourth COVID-19 vaccine dose on a diverse Brazilian population with regard to the third vaccine dose and SARS-CoV-2 variants.

## RESULTS

### Composition and preliminary serological analysis of the study population

**Fig 1:**
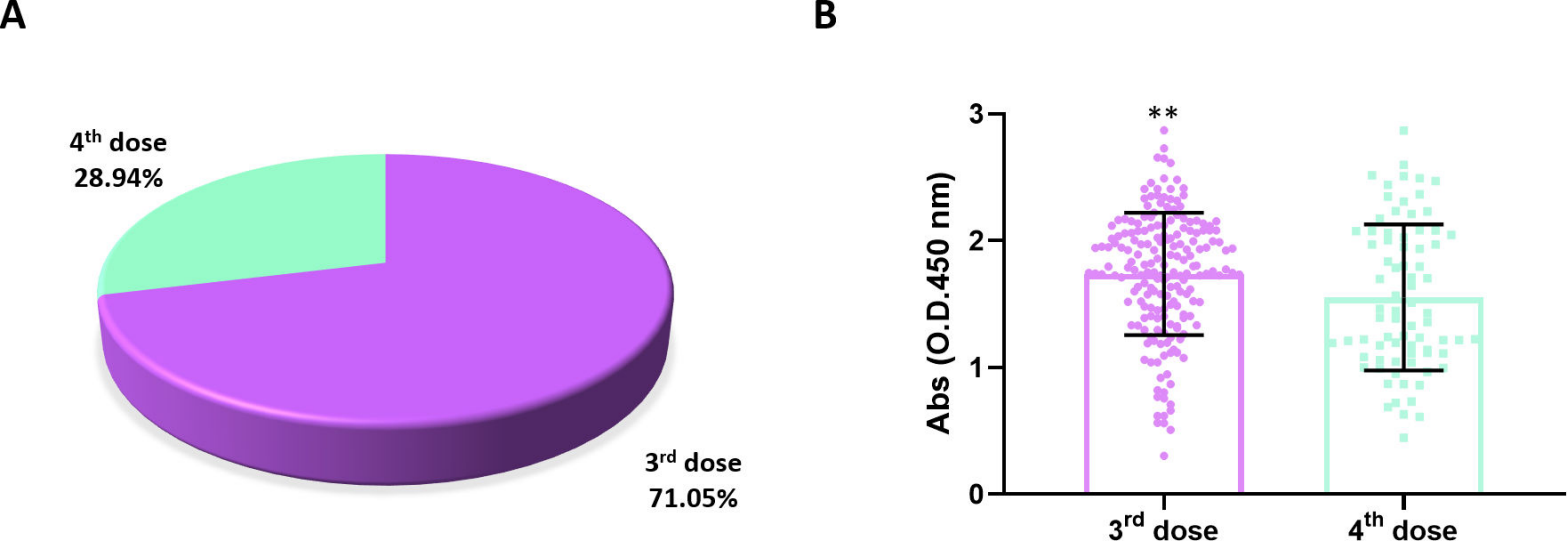
Composition and preliminary serological analysis of the study population. (A) The main population was composed of 266 volunteers who were vaccinated against COVID-19: 189 and 77 individuals received three or four doses, respectively. (B) Serum levels of specific antiviral antibodies [represented as optical densities (ODs)] of volunteers who were vaccinated with three or four doses were obtained by enzyme‐linked immunosorbent assay (ELISA) and analyzed using the Mann–Whitney *U* test. The group immunized with three doses presented significantly higher serum levels of specific antibodies (*P* ≤ 0.001). Medians of groups were compared using the Mann–Whitney *U* test. (B) Statistical significance was set as *P* ≤ 0.05. Statistical power was set to be at least 80%. Standard deviations (SDs) are given as error bars.

The main study population was comprised of 266 volunteers who received either three (189) or four (77) vaccine doses (Fig. 1A). None of the volunteers who received the third or fourth doses were infected with SARS-CoV-2 for at least 1 year before the study period, as determined by the lack of registered evidence of illness. As evidenced by enzyme‐linked immunosorbent assay (ELISA) results, individuals who received three vaccine doses had significantly higher serum levels of antiviral-specific antibodies (*P* < 0.001) than individuals who received four vaccine doses (Fig. 1B). This result indicates that the serum levels of antibodies capable of specifically recognizing SARS-CoV-2 structural proteins in ELISA were not boosted with the fourth vaccine dose.

### Serological analyses according to the time interval between the last two vaccine doses

**Fig 2:**
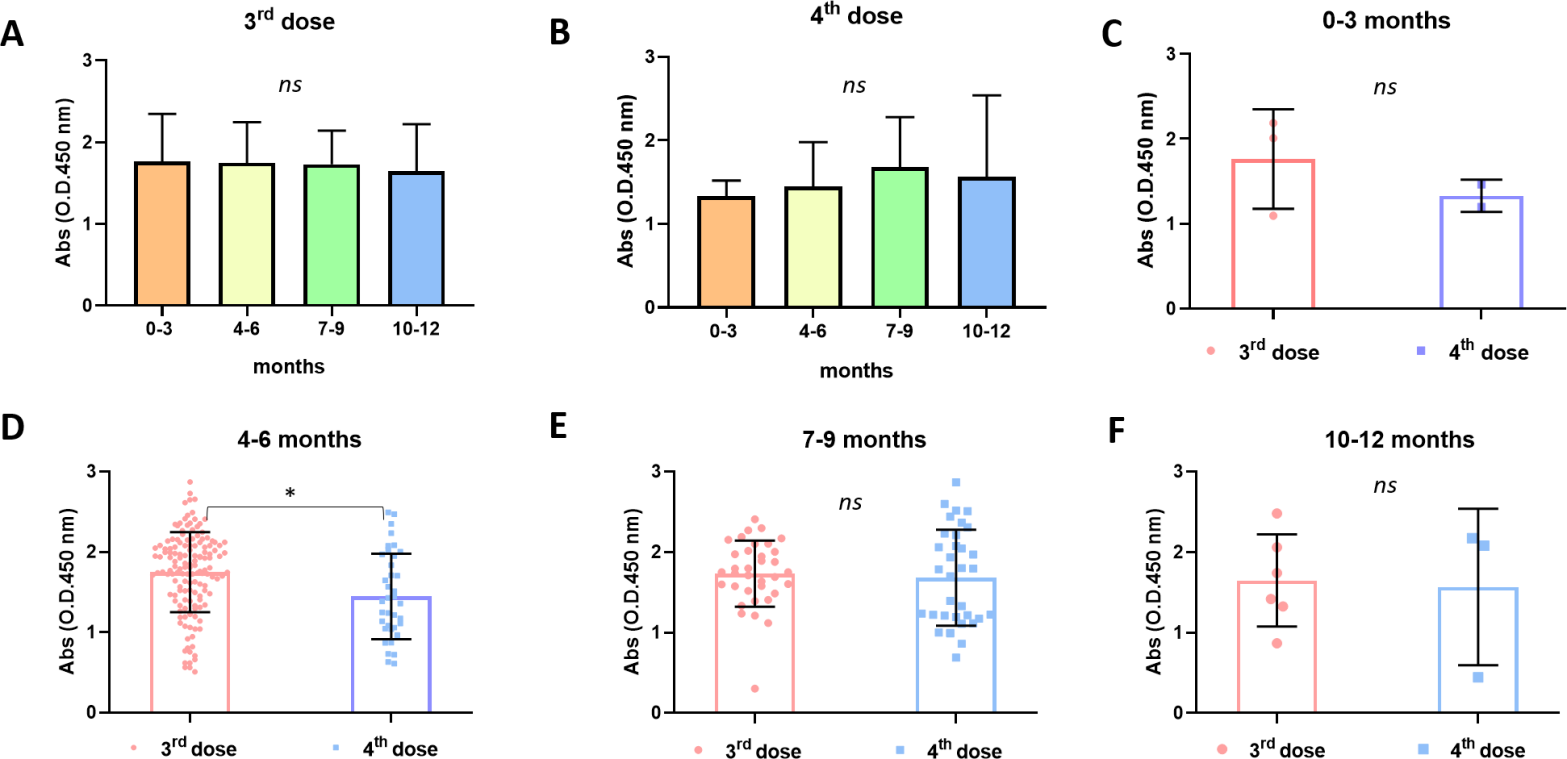
Serological analyses according to the time interval between the last two doses. Serum levels of specific antibodies were shown to be statistically indistinguishable when the time interval between the second and third doses (A) and the third and fourth doses (B) was 0–3, 4–6, 7–9, or 10–12 months. When serum levels of specific antibodies obtained from volunteers who received three or four vaccine doses were compared according to the time interval between the last two doses, no difference was seen with a 0- to 3-month interval (C). Volunteers who received three doses presented higher serum levels of specific antibodies than those who received four doses when the time interval between the last two doses was 4–6 months (D). No difference was noted when the time interval between the last two doses was 7–9 (E) or 10–12 mo (F). Medians of groups were compared using the Kruskal–Wallis test followed by Dunn’s multiple comparisons (A and B) or the Mann–Whitney *U* test (C–F). Statistical significance was set as *P* ≤ 0.05. Statistical power was set to be at least 80%. Standard deviations (SDs) are given as error bars.

Further analysis showed that the serum levels of SARS-CoV-2 specific antibodies were statistically indistinguishable when the time interval between the second and third vaccine doses (Fig. 2A) or between the third and fourth doses (Fig. 2B) was 0–3, 4–6, 7–9, or 10–12 months. On comparison of the serum levels of specific antibodies obtained from individuals who received three or four vaccine doses to the time interval between the last two doses, no difference was observed in individuals with a 0- to 3-month interval between the last two doses (Fig. 2C). In contrast, individuals vaccinated with three doses displayed significantly higher serum levels of specific antibodies than those who received four doses when the time interval between the last two doses was 4–6 months (Fig. 2D). However, no statistically significant differences were noted when the time interval between the last two doses was 7–9 (Fig. 2E) or 10–12 months (Fig. 2F). In addition, no sustained tendency for an increase or decrease in serum-specific antibody levels was noted with respect to the time of sample collection following the last vaccine dose (Fig. S1). Collectively, these results indicate that there was no sustained significant change in the serum levels of antibodies capable of recognizing viral structural proteins according to the time interval between the last two doses. These results indicated that the fourth vaccine dose did not boost the serum levels of antiviral antibodies with respect to the third dose, regardless of the interval between the last two doses.

### Serological analyses according to the immunization regimen and different vaccine formulations

**Fig 3:**
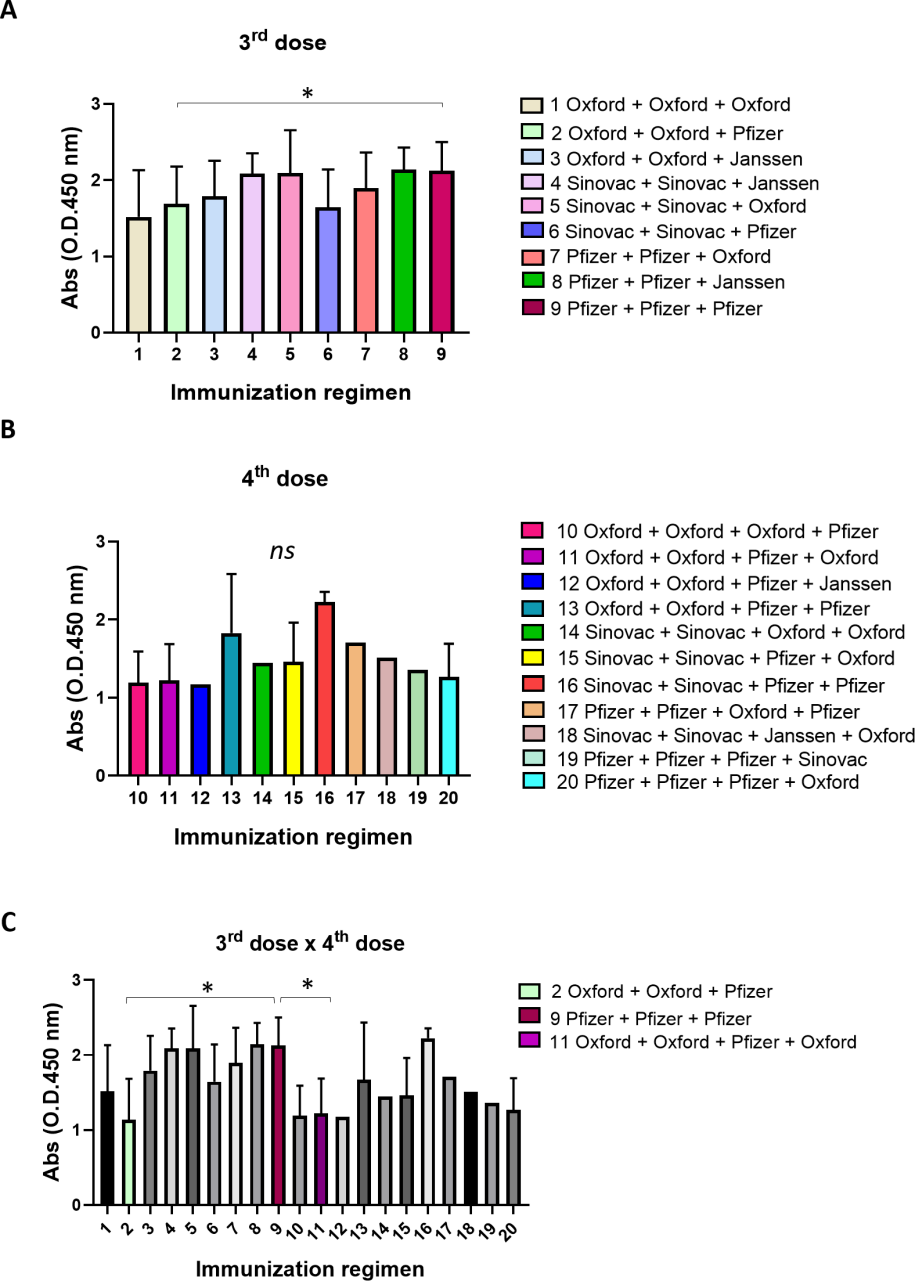
Serological analyses according to the immunization regimen. Serum levels of specific antiviral antibodies were measured in samples from volunteers who were subjected to different immunization regimens composed of three (A) or four (B) vaccine doses. Volunteers who received three doses of the Pfizer vaccine presented with higher serum levels of antiviral antibodies than those who received two doses of the Oxford vaccine, followed by a third dose of the Pfizer vaccine. In addition, volunteers who received three doses of the Pfizer vaccine presented with higher serum levels of antiviral antibodies than those who received two initial doses of the Oxford vaccine, followed by a third dose with the Pfizer vaccine and a fourth dose with the Oxford vaccine (C). Medians of groups were compared using the Kruskal–Wallis test followed by Dunn’s multiple comparisons. Statistical significance was set as *P* ≤ 0.05. Statistical power was set to be at least 80%. Standard deviations (SDs) are given as error bars.

Serum levels of antiviral antibodies were measured in samples from individuals who received three or four vaccine doses under different immunization regimens. As shown in Fig. 3A, individuals who received three doses of the Pfizer vaccine presented higher serum levels of antibodies capable of recognizing viral structural proteins than those who received two doses of the Oxford vaccine, followed by a third dose of the Pfizer vaccine. No differences were observed in the serum levels of antiviral antibodies in individuals who received four vaccine doses with respect to the different immunization regimens (Fig. 3B). In addition, individuals who received three doses of the Pfizer vaccine presented with higher serum levels of SARS-CoV-2-specific antibodies than those who received an immunization regimen composed of two initial doses of the Oxford vaccine, followed by a third dose of the Pfizer vaccine and a fourth dose of the Oxford vaccine (Fig. 3C). These results indicate that the immunization regimen composed of three doses of the Pfizer vaccine was probably related to the differences in the serum levels regarding the 4–6 months’ time interval between the last two doses seen in Fig. 2D.

### Antibody neutralization of SARS-CoV-2 variants with respect to vaccination history

**Fig 4:**
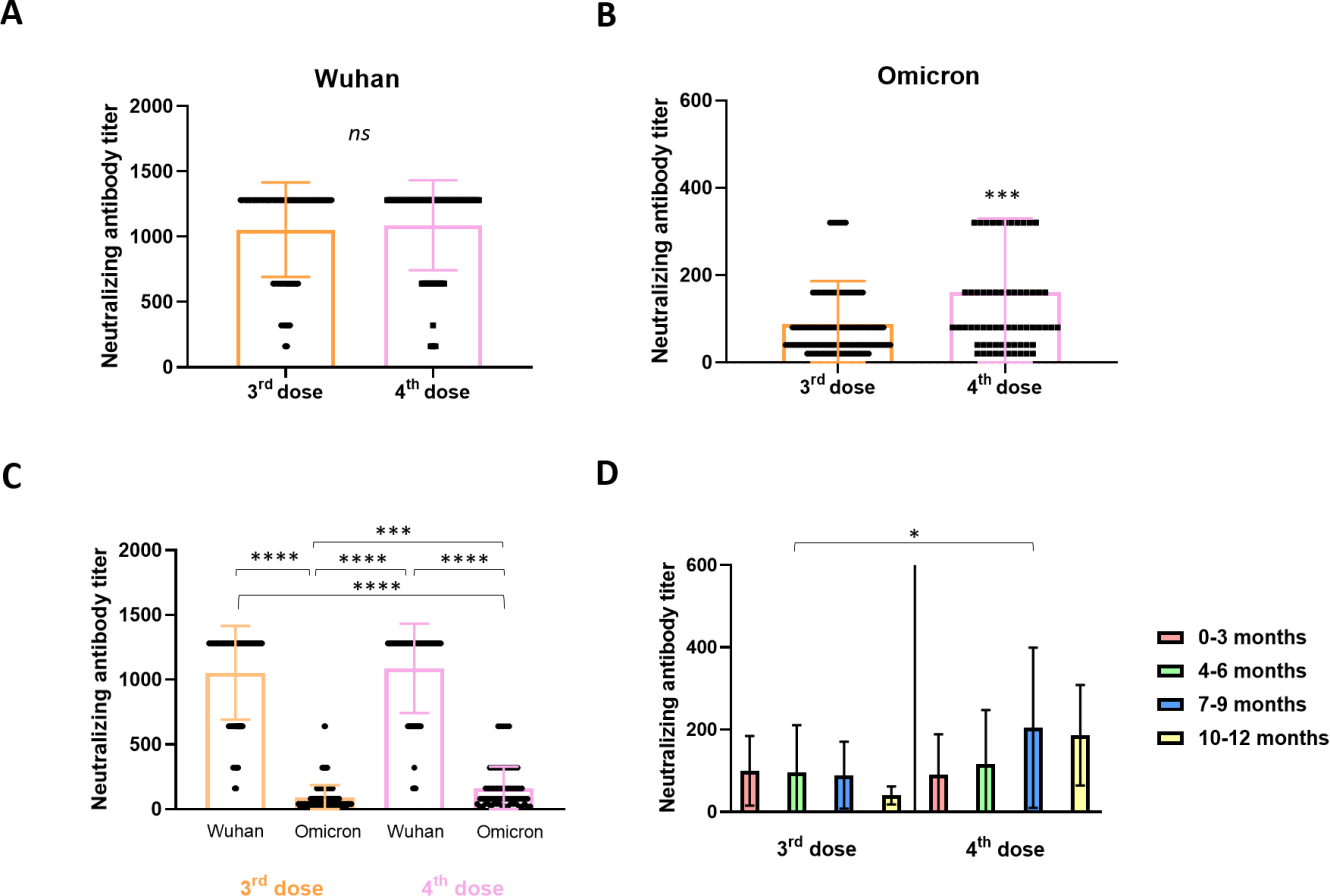
Serum neutralization levels against SARS-CoV-2 Wuhan and Omicron variants. Serum samples from volunteers with different vaccine histories were subjected to cytopathic effect‐based virus neutralization test (CPE‐VNT) using Wuhan and Omicron SARS‐CoV‐two variants. No difference was observed with the Wuhan virus between samples from volunteers who received three or four doses (A). Significantly higher titers of Omicron neutralizing antibodies were measured in samples from volunteers immunized with four vaccine doses (B). Similar results were observed when all groups were analyzed together (C). When samples were grouped according to the time interval between the last two doses, neutralizing antibody titers from volunteers with a time interval of 7–9 months between the third and fourth doses were significantly higher than those observed in volunteers with a time interval of 4–6 months between the second and third doses (D). Medians of groups were compared using the Mann–Whitney *U* test (A and B) or the Kruskal–Wallis test followed by Dunn’s multiple comparisons (C and D). Statistical significance was set at *P* ≤ 0.05. Statistical power was set to be at least 80%. Some of the dots indicating neutralization titers seem like bars because they indicate the same values: some volunteers presented the same titers. Standard deviations (SDs) are given as error bars.

In order to check whether the quantitative analysis directly reflected the serum neutralization capacity against the Wuhan–WT and/or the Omicron VOC, samples from volunteers with different vaccination histories were subjected to a cytopathic effect‐based virus neutralization test (CPE‐VNT). As shown in Fig. 4A, no statistical difference was observed between the Wuhan-NAb titers in samples from individuals vaccinated with three or four doses. In contrast, significantly higher NAb titers against the Omicron VOC were observed in samples from volunteers immunized with the four vaccine doses (Fig. 4B). Similar results were observed when all groups were analyzed together (Fig. 4C). When samples were grouped according to the time interval between the last two doses, NAb titers of samples from individuals with a time interval of 7–9 months between the third and fourth doses were significantly higher than those from individuals with a time interval of 4–6 months between the second and third doses (Fig. 4D). These results indicate that despite the lower SARS-CoV-2-specific antibody levels as detected by ELISA, the fourth vaccine dose significantly increased NAb levels against the SARS-CoV-2 Omicron variant.

### Conservation analysis of NAb epitopes in different Omicron subvariants circulating in the study area

**Fig 5:**
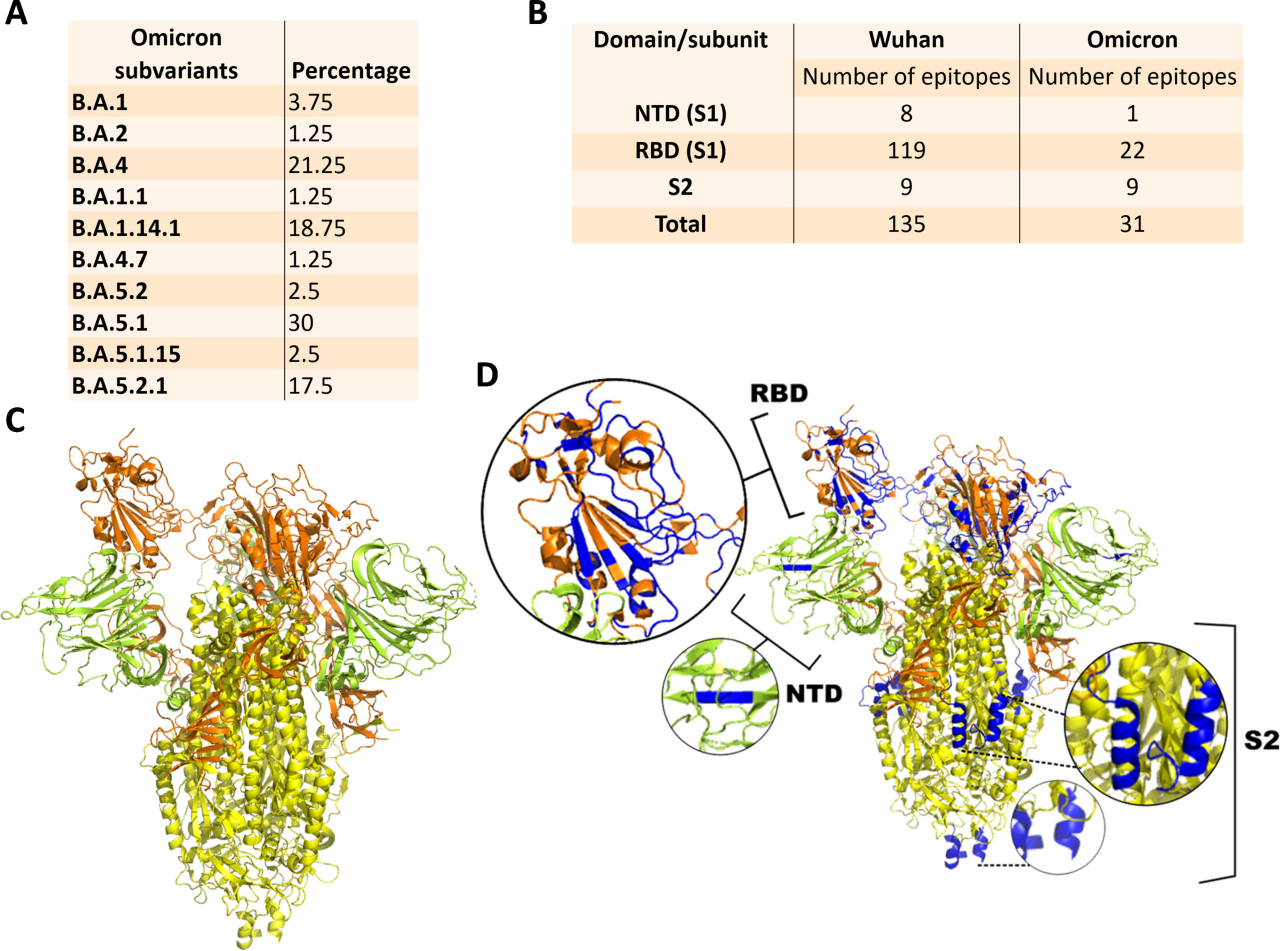
Detection of SARS-CoV-2 variants and analysis of epitope conservation. Only Omicron subvariants were detected in the study area during the study period of study, according to genomic analysis. Conservation of known neutralizing epitopes among viruses detected in the study area was assessed. Percentages of Omicron subvariants detected in COVID-19-positive patients in the study area during the period of study (A). Number of known neutralizing epitopes in the Spike protein of the Wuhan and Omicron subvariants detected in the present study (B). Image representing the trimer of the SARS-CoV-2 Spike protein: subunit 2 (S2) is shown in yellow; N-terminus domain (NTD) is shown in green; receptor-binding domain (RBD) is shown in orange (C). Location of the 100% conserved neutralizing epitopes in the SARS-CoV-2 Spike protein (D). Conserved epitopes are shown in blue. Zoomed images of conserved epitopes in the RBD and NTD are shown.

To better characterize the exposure to VOCs during the study period, we analyzed samples collected from patients with flu-like symptoms and detected SARS‐CoV‐2 in 96 of the 457 samples. Suitable amplicons were generated for 82 samples and were subjected to genome sequencing. After analysis of genome coverage (at least ≥20×), 57 samples were deposited in the GISAID‐EpiCoV. Analyses using the Pangolin web application version indicated that only subvariants of the SARS-CoV-2 Omicron VOC were detected during the study period, as shown in Fig. 5A. Subvariants B.A.5.1, B.A.4, B.A.1.14.1, and B.A.5.2.1 were predominant in the present study. Conservation analysis of epitopes of S protein of a Wuhan virus and a local Omicron subvariant recognized by NAbs revealed that most of the immune targets were abrogated in viruses circulating in the study area during the study period (Fig. 5B). Epitopes located in the N-terminal domain of the S protein were reduced from eight in Wuhan viruses to one in the locally circulating viruses. In addition, the number of epitopes located in the receptor-binding domain was reduced from 119 in Wuhan viruses to 22 in locally circulating viruses. Only the epitopes located in subunit 2 (S2) of the S protein were completely conserved. Using the locations of the spike subunits and domains (Fig. 5C), we identified a few conserved epitopes in the entire protein structure. There was a reduction from 135 epitopes in the Wuhan viruses to 31 in the locally circulating viruses (Fig. 5D and B). Collectively, these results indicate that the study populations were exposed to viruses that conserved the limited number of epitopes for NAbs with regard to those presented to their immune systems by vaccines based on the Wuhan–WT SARS-CoV-2.

## DISCUSSION

Immunization programs have shown success in controlling COVID-19 worldwide, especially in severe and lethal cases. In our study area, implementation of the original vaccination regimens (two doses) and administration of additional doses reduced the number of cases by half, reduced the intensive care unit occupancy rate, and drastically reduced the number of deaths (see Table 1). Such progress was achieved by vaccines based on the wild-type SARS-CoV-2, even when Omicron subvariants were dominant in the study area during the study period. We have previously shown that a third dose of a SARS-CoV-2 wild-type-based vaccine is capable of conferring protection against Omicron subvariants by eliciting NAbs (15). It is also important to understand the impact of the fourth vaccine dose on the fight against COVID-19. However, studies showing the immune response and efficacy of the fourth vaccine dose are based on restricted populations that are mainly composed of elderly, and/or immunocompromised subjects, and healthcare workers. To understand the impact of the fourth dose in the general population, we enrolled healthy volunteers of various ages and occupations. In addition, we monitored viruses circulating in the study area to better understand the antigens being presented to the study population. It is important to highlight that this study was conducted before the availability and recommendation of a fifth dose based on bivalent vaccines, which include a component of the original virus strain, and a component of the newest viruses: subvariants of the Omicron VOC (BA.1, BA.4, and BA.5) (25, 26).

In contrast to what was observed with administration of the third dose (14), we did not see a boost in serum levels of antibodies capable of recognizing viral structural proteins after the fourth dose compared to that observed with the third dose. Surprisingly, we found that the group of immunocompetent individuals, belonging to different genders, ages, and races enrolled in the present study, that were immunized with up to three vaccine doses had higher levels of antiviral antibodies than those immunized with four vaccine doses. In addition, deeper investigation revealed that such difference could be attributed to the immunization regimen with three doses of the RNA-based Pfizer vaccine. As reported previously, such a homologous vaccine regimen is significantly better at inducing antiviral antibodies than other immunization strategies (27, 28), even in immunocompromised individuals (29).

Compared to the higher antibody levels elicited by the three dose-based regimens, we found that the fourth dose increased the serum levels of NAbs capable of neutralizing the Omicron variant. This result indicated that there was a refinement of the humoral immune response to neutralize the virus, possibly due to the expansion of the repertoire of broad B-lymphocyte memory cells against highly conserved epitopes, as was previously observed with multiple contacts with SARS-CoV-2 antigens (30). This explanation is supported, at least in part, by our findings that most of the epitopes recognized by NAbs present in the Wuhan virus were not present in the virus variants detected during the study period of the present study. There was a reduction of 135 epitopes in the Wuhan S protein to 31 epitopes in the Omicron subvariants detected in the study area. Thus, it is reasonable to assume that antibodies capable of neutralizing the Omicron variant are driven by these few conserved epitopes. It is also important to highlight that these epitopes are fully conserved, and not a single amino acid was changed from the Wuhan virus to the Omicron viruses. As the study populations were exposed to viruses that conserved a rather reduced number of epitopes recognized by NAbs compared to those presented to their immune systems by vaccines based on the Wuhan–WT SARS-CoV-2, the explanation given here remains plausible.

Although the above explanations could explain the observed findings, the molecular mechanisms remain to be investigated further. Conservation of 31 epitopes may be related to the key biological functions of the spike protein, especially the maintenance of structural stability and binding to the host cell receptor. A similar limit in mutations was previously reported in flaviviruses, with the most conserved epitopes recognized by NAbs concentrated in structures with key biological functions (25, 26). In addition, the lack of an increase in serum levels of antibodies capable of recognizing viral proteins in ELISA, in contrast to the increase in serum levels of NAbs, may also be due to the immunodominance of a few conserved epitopes. Further studies are required to elucidate these gaps. Nonetheless, we presented robust data supported by proper statistical analyses, showing that the fourth vaccine dose improved the serum levels of antibodies capable of neutralizing the Omicron variant in a population that was exposed to viruses, conserving a limited number of epitopes targeted by their antibodies.

### Limitations

The sample size of the main study population in this study was not representative of the city’s population. We were unable to achieve the intended sample size because of low enrollment. We had also discrepancies in the numbers of volunteers representing different time frames between the last two doses. There was a dominance of samples representing 4–6 months regarding the third dose group, and 7–9 months regarding the fourth dose group. In addition, serum analyses by ELISA were based only on solid-phase antigens from the Wuhan–WT SARS-CoV-2. Moreover, we did not perform neutralization assays with respect to the Omicron subvariants. We used only an Omicron (BA.1) viral strain. Furthermore, we did not have samples from volunteers who received four doses of the Pfizer vaccine, a homologous four dose-based regimen, for comparison with the three dose-based regimens.

### Conclusions

In the present study, we presented robust data supported by statistical analyses (minimal power of 80% for statistical significance), which showed that the fourth vaccine dose improved the serum levels of antibodies capable of neutralizing the Omicron variant in a population exposed to viruses that conserved a limited number of epitopes targeted by NAbs. Our data strongly support the conclusion that four doses of COVID-19 vaccines based on the Wuhan–WT SARS-CoV-2 can contribute to a reduction in susceptibility to the Omicron variant and its sub-variants. It is important to highlight that the Brazilian context is representative for the broader society, because there are subgroups of vaccination regimens that represent the use of homologous, and heterogeneous schemes, with vaccine platforms based on mRNA, adenovirus vector, or inactivated viruses. In all cases, individuals who received four vaccine doses seem to have benefited from a humoral immune response based on NAbs that could prevent disease caused by Omicron subvariants that were circulating in the study area in the study period. Our conclusion is that the fourth COVID-19 vaccine dose increased the neutralizing antibody response against SARS-CoV-2 Omicron variant in a diverse Brazilian population.

## MATERIALS AND METHODS

### Study design and ethics

This retrospective observational study included two populations from Barreiras, Bahia, Brazil. Data and samples collected from May to September 2022 were used in this study. The main population consisted of 266 healthy volunteers (77 males and 189 females) with diverse occupations (students, health professionals, teachers, government employees, and retirees) aged between 18 and 81 years who received three (*n* = 189) or four (*n* = 77) COVID-19 vaccine doses of Sinovac-CoronaVac, Oxford/Astrazeneca (AZD1222 or ChAdOx1-S), Janssen (Ad26.COV2.S), or Pfizer (BNT126b2, RNA-based vaccine), as homologous or heterologous regimens. The group of three doses consisted of 124 females with average age of 39 ± 16.42 years, and 65 males with average age of 38 ± 17.13 years. The group of fourth dose was composed of 65 females with average age of 37 ± 15.11 years, and 12 males with average age of 36 ± 13.95 years (Supplementary Material 1). We collected blood samples to obtain immunological profiles based on ELISA and neutralization assays, as well as their COVID-19 vaccination, and COVID-19 (disease) histories, according to local health authorities’ official data, as previously described (14). The second population consisted of 457 patients with flu-like symptoms (199 males and 258 females), aged between 4 and 84 years, and were enrolled during a COVID‐19 epidemic that occurred in the city in the period of study. This population differed from the main population because most enrolled individuals did not receive the third and/or the fourth doses or in some cases even the original vaccine regimen (two doses). Additionally, nasopharynx swab samples were collected and used for molecular diagnosis based on one-step reverse transcription, followed by real-time polymerase chain reaction (RT-PCR) and viral genome sequencing. The study complied with the relevant ethical and biosafety guidelines. Ethical approval was obtained from the Institutional Ethics Committee of the Federal University of Western Bahia (CAAE 40779420.6.0000.8060). All the procedures and possible risks were explained to the volunteers. Informed consent was obtained from all study participants.

### Enzyme-linked immunosorbent assay

Serum samples were analyzed using the EIE COVID-19 IgG N/S kit (Bio-Manguinhos, Fiocruz, Rio de Janeiro, Brazil) according to the manufacturer’s instructions, as described previously (14, 15). Serum levels of antibodies specific to the SARS-CoV-2 structural proteins spike (S) and nucleoprotein (N) were defined according to the optical density values. Briefly, an ELISA with solid-phase bound N and S recombinant antigens was performed using serum samples from volunteers. Kit controls and samples were added to the wells after dilution (1:101) with the kit diluent. After incubation for 30 min at 37°C, plates were washed five times with kit washing buffer. Subsequently, the diluted (1:100) conjugate provided in the kit was added to each well and the plates were further incubated for 30 min at 37°C. The plates were then washed five times, and the reaction was initiated by the addition of the developing solution to the wells. After incubation at room temperature for 10 min, the reaction was terminated with 2 M H_2_SO_4_. The absorbance was measured at 450 nm.

### Cell culture and SARS-CoV-2 propagation

The experiments involving SARS‐CoV‐2 were carried out in laboratory biosafety level 3 (BSL3) facilities, in accordance with the recommendations of the World Health Organization (WHO). African Green monkey kidney cells Vero E6 (ATCC CRL‐1586) and Vero CCL‐81 (ATCC CCL‐81) were maintained according to the recommendations of ATCC. Vero E6 cell monolayer was infected with SARS‐CoV‐2 variants to propagate a viral stock. The following SARS‐CoV‐2 strains used in the present study: (i) wild-type SARS‐CoV‐2 (Wuhan strain—WT) (GISAID: EPI_ISL_2499748), a kind gift from Dr. José Luiz Proença‐Módena (University of Campinas—UNICAMP, Campinas, SP, Brazil); and (ii) Omicron variant (GISAID: EPI_ISL_6794907), a kind gift from Dr. Edison L. Durigon (University of São Paulo, USP, São Paulo, SP, Brazil). The SARS‐CoV‐2 viral stocks were subjected to titration [in tissue culture infectious dose (TCID) 50 /mL], as described previously (15), and were used for viral neutralization tests.

### Cytopathic effect‐based virus neutralization test for SARS-CoV-2 WT and Omicron variants

The CPE‐VNT assay was performed in a BSL3 laboratory, according to WHO recommendations. Nab titers against SARS-CoV-2 variants were measured as described previously (15). Briefly, cell monolayers (5 × 10^4^ Vero CCL‐81 cells/well) in 96‐well culture plates were exposed to 1 × 10^3^ TCID50/mL of SARS‐CoV‐2 Wuhan strain—WT or Omicron variants that were previously incubated with 1:20–1:1,280 twofold diluted, heat‐inactivated human serum samples, in a final volume of 150 µL. After 72 h of incubation, the plates were evaluated microscopically for the presence of characteristic SARS‐CoV‐2 CPEs. The absence of CPEs in the 1:20 diluted sample was considered as a positive result for the presence of neutralizing antibodies against SARS‐CoV‐2.

### RNA extraction and RT-PCR

Nucleic acid extraction from nasopharyngeal samples was performed using the Extracta Kit—Viral RNA and DNA (MVXA‐ P016FAST) (Loccus, Brazil) using an Extracta32 instrument (Loccus) as previously described (15). Laboratory diagnosis was based on one-step reverse transcription followed by RT-PCR using the INFA/INFB/SC2 kit (Bio-Manguinhos, Brazil) as described previously (15).

### SARS-CoV-2 genome sequencing

Viral RNA was extracted as described above. Complementary DNA and PCR products were obtained using the Midnight RT-PCR Expansion kit (EXP‐MRT001) (Oxford Nanopore Technologies, UK) as per the manufacturer’s instructions, and generated amplicons of ~1,200 bp that overlapped the entire SARS‐CoV‐2 genome. Of the SARS‐CoV‐2‐positive nasopharynx swab samples (*n* = 96), only those that resulted in the successful generation of amplicons (*n* = 82) were subjected to genome sequencing using next-generation sequencing on the Oxford Nanopore MinIon platform (Oxford Nanopore Technologies). The Rapid Barcoding Kit 96 (SQK‐RBK110.96) (Oxford Nanopore Technologies) was used to barcode the pool of multiple samples, which was then purified, and 800 ng was used for library preparation and sequencing using the Oxford Nanopore MinION SpotON Flow Cells R9 version (Oxford Nanopore Technologies), following the manufacturer’s instructions. Sequencing was performed using the so‐called rapid precision base in MinKNOW software according to the defined protocol [Community‐Protocol‐PCR tiling of SARS‐CoV‐2 virus‐rapid barcoding and Midnight RT PCR Expansion (SQK‐RBK110.96 and EXP‐MRT001)] (nanoporetech.com). RAMPART (https://artic.network/ncov-2019) was used to monitor the sequencing run in real time to estimate the depth of coverage (20×) across the genome for each barcode (https://artic.net/wall). Analysis and consensus generation were performed according to the pipeline proposed by the ARTIC Network using the Medaka protocol (artic.network/ncov-2019/ncov2019-bioinformatics-sop.html). New full-genome sequences of SARS‐CoV‐2 obtained in the present study were submitted to the Pangolin web application version v4.1.3 and pangolin‐data version v1.17, available at https://pangolin.cog-uk.io/. Consensus genomes with coverage of ≥20× (*n* = 57) were deposited in the Global Initiative on Data Sharing Avian Influenza EpiCoV (GISAID‐EpiCoV) database (see Supplementary Material 2 for details).

### Statistical analyses

The median serum levels of specific and neutralizing antibodies after the third or fourth dose were compared using the Mann–Whitney *U* test. For comparison between multiple (>2) groups, the Kruskal–Wallis test, followed by Dunn’s multiple comparisons, was used. In all cases, statistical significance was set at *P* ≤ 0.05. Statistical power was set to be at least 80%.

### Immunoinformatics

In the present study, data sets comprised of the amino acid sequences of the spike protein (S) of the Wuhan–WT and Omicron SARS-CoV-2 were built (Supplementary Material 3). The Wuhan data set was composed of viral sequences generated in different continents, retrieved from the National Center for Biotechnology Information (https://www.ncbi.nlm.nih.gov/), and enriched with genomic sequences from GISAID (https://gisaid.org/). The criteria for selecting the sequences were as follows: (i) complete sequences; and (ii) absence of unidentified amino acids. The Omicron data set was composed of sequences generated in the present study from viruses circulating during the study period. The Wuhan data set consisted of 28 amino acid sequences, whereas the Omicron database consisted of 22 amino acid sequences. In addition, the amino acid sequences of the real epitopes for the neutralizing antibodies were retrieved from the Immune Epitope Database (IEDB) (https://www.iedb.org/). The data set is comprised of 425 epitopes for neutralizing antibodies (Supplementary Material 4). The IEDB conservation analysis tool (http://tools.iedb.org/conservancy) was used to determine epitope conservation among all SARS-CoV-2 Spike protein sequences, as described previously (31, 32). In the present study, only 100% conserved epitopes were considered. To localize highly conserved epitopes, a spike protein model retrieved from the Protein Data Bank (PDB- https://www.rcsb.org/search/advanced/sequence) was used. Fully conserved epitopes were identified in a 3D model of the SARS-CoV-2 spike protein (DOI: 10.2210/pdb7dk3/pdb) (33) using PyMol (https://pymol.org/2/), as described previously (32, 34).

## Data Availability

Data sets used in this study are available as supplemental material. Accession numbers are cited in data sets. Results of analyses are shown in the main text, tables, and figures.

## SUPPLEMENTAL MATERIAL

Supplementary Figure 1 spectrum.02857-23-s0001.tif Supplementary Figure 1 - Serological analyses according to the time of sample collection following the last vaccine dose.

Supplementary Material 1 spectrum.02857-23-s0002.xlsx Supplementary Material 1 - Demographic data of the study population.

Supplementary Material 2 spectrum.02857-23-s0003.xlsx Supplementary Material 2 - Metadata of viral genomic sequences.

Supplementary Material 3 spectrum.02857-23-s0004.docx Supplementary Material 3 - Amino acid sequences of the spike protein (S) of the Wuhan-WT and Omicron SARS-CoV-2.

Supplementary Material 4 spectrum.02857-23-s0005.docx Supplementary Material 4 - Amino acid sequences of 425 epitopes for neutralizing antibodies.

Supplementary Figure 1 legend spectrum.02857-23-s0006.docx Legend for Supplementary Figure 1.

ASM does not own the copyrights to Supplemental Material that may be linked to, or accessed through, an article. The authors have granted ASM a non-exclusive, world-wide license to publish the Supplemental Material files. Please contact the corresponding author directly for reuse.
