# Supplementary figures and images for: The fourth COVID-19 vaccine dose increased the neutralizing antibody response against the SARS-CoV-2 Omicron (B.1.1.529) variant in a diverse Brazilian population
Source: Microbiol Spectr. 2023 Nov 1;11(6):e02857-23. doi: 10.1128/spectrum.02857-23 (PMC10714775; doi:10.1128/spectrum.02857-23)

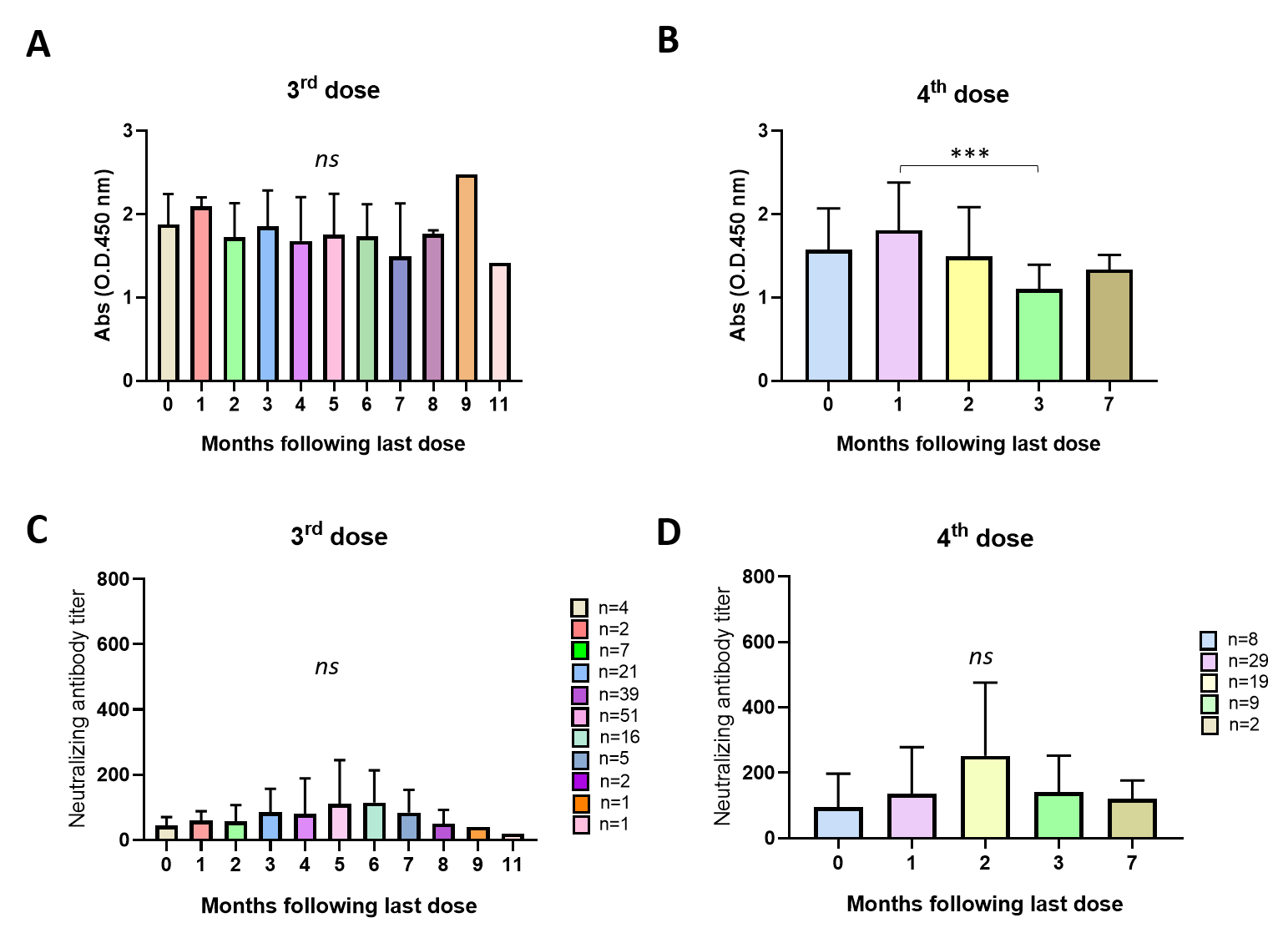

Supplement: Supplementary Figure 1 — Serological analyses according to the time of sample collection following the last vaccine dose. [file spectrum.02857-23-s0001.tif]
