# Supplementary material for: The fourth COVID-19 vaccine dose increased the neutralizing antibody response against the SARS-CoV-2 Omicron (B.1.1.529) variant in a diverse Brazilian population
Source: Microbiol Spectr. 2023 Nov 1;11(6):e02857-23. doi: 10.1128/spectrum.02857-23 (PMC10714775; doi:10.1128/spectrum.02857-23)
Supplement: Supplementary Material 4 — Amino acid sequences of 425 epitopes for neutralizing antibodies. [file spectrum.02857-23-s0005.docx]

Supplementary Material 4 – Amino acid sequences of 425 epitopes for neutralizing antibodies.

> DESCONTINUOUS EPITOPES

Y369,N370,S371,A372,F374,F377,K378,C379,Y380,G381,V382,S383,P384,T385,K386,L390,F429,T430,F515,E516,L517

A475,V483,F486,S494

Y369,N370,A372,F374,T376,F377,K378,Y380,V382,P384,T385,K386,D389,L390,F392,D428,F429,T430,F515,L517,H519

R346,K444,G446,G447,N448,Y449,N450,L452,V483,E484,G485,F490,S494

R403,Q409,T415,G416,K417,D420,Y421,L455,F456,R457,K458,S459,N460,Y473,Q474,A475,G476,S477,F486,N487,Y489,F490,Q493,Y495,G496,Q498,N501,G502,Y505

R403,D405,E406,R408,Q409,T415,G416,K417,D420,Y421,L455,F456,R457,K458,N460,Y473,Q474,A475,G476,S477,F486,N487,Y489,Q493,Y495,G502,Y505

G446,Y449,E484,G485,F486,Y489,F490,L492,Q493,S494,G496,Q498,N501,Y505

Y369,N370,S375,T376,F377,K378,C379,Y380,G381,V382,S383,P384,T385,K386,L390,F392,D428,T430,E516,L517

R403,D405,R408,T415,G416,K417,D420,Y421,Y453,L455,F456,R457,K458,S459,N460,Y473,Q474,A475,G476,S477,F486,N487,Y489,Q493,S494,Y495,G496,Q498,T500,N501,G502,Y505

R403,D405,T415,G416,K417,D420,Y421,Y453,L455,F456,R457,K458,N460,Y473,A475,G476,S477,F486,N487,Y489,Y495,N501,Y505

Y144,Y145,H146,K147,K150,W152,H245,R246,S247,Y248,L249

A372,F374,C379

F374,S375,T376,F377,C379,F392,D427,E516

L455,A475,G502

Y449,Y453,L455,F456,E484,G485,F486,Y489,F490,L492,Q493,S494

Y369,S375,F377,K378,C379,Y380,G381,V382,S383,P384,T385,K386,F392,P412,G413,D427,D428,F429,L517

Y369,N370,F374,S375,T376,F377,K378,C379,Y380,G381,V382,S383,P384,T385,K386,L390,R408,D428,T430,L517,L518

N334,L335,P337,G339,E340,N343,A344,T345,R346,K356,R357,S359,N360,C361,L441

G446,Y449,Y453,L455,F456,A475,G476,S477,T478,G485,F486,N487,Y489,Q493,Y495,Q498,N501,Y505

R346,F347,S349,Y351,K444,G446,G447,N448,Y449,N450,Y451,L452,T470,E484,F490,L492,Q493,S494

L455,Y473,A475,G476,S477,E484,G485,F486,N487,C488,Y489

G446,Y449,N481,G482,V483,E484,G485,F486,F490,S494

K444,G446,Y449,N450,L452,N481,G482,V483,E484,G485,F490

N334,L335,P337,G339,E340,N343,A344,T345,R346,K356,R357,S359,C361,L441

R403,D405,T415,G416,K417,D420,Y421,Y453,L455,F456,R457,K458,N460,Y473,Q474,A475,G476,S477,T478,F486,N487,Y489,Q493,N501,Y505

R403,T415,G416,K417,D420,Y421,Y453,L455,F456,R457,K458,N460,Y473,A475,G476,S477,F486,N487,Y489,Q493,S494,Y495,G496,Q498,T500,N501,G502,V503,Y505

R403,T415,G416,K417,D420,Y421,Y453,L455,R457,K458,S459,N460,Y473,Q474,A475,G476,S477,F486,N487,Y489,Q493,Q498,T500,N501,G502,V503,Y505

R403,V445,G446,Y449,Y453,L455,F456,N487,Y489,Q493,Y495,G496,Q498,P499,T500,N501,G502,Y505

T415,G416,K417,D420,Y421,Y453,L455,F456,R457,K458,S459,N460,Y473,Q474,A475,G476,S477,F486,N487,Y489,Q493,S494,Y495,G496,Q498,T500,N501,G502,Y505

Y369,N370,F377,K378,C379,Y380,G381,V382,S383,P384,T385,K386,N388,L390,F392,P412,Q414,D427,D428,F429,T430,L517

Y369,N370,S371,A372,F374,S375,T376,F377,K378,C379,S383,P384,T385,R408,Q414

D420,A475,N487

E484,F490

F486,N487

S443,V445,G446,G447,Y449,G496,Q498,P499,T500

T376,K378,R408,K417

R346,K444,G446,G447,N448,Y449,N450,L452,V483,E484,G485,F490,S494

R403,T415,G416,K417,D420,Y421,Y453,L455,F456,R457,K458,S459,N460,Y473,Q474,A475,G476,S477,F486,N487,Y489,Q493,G502,Y505

V445,G446,Y449,F456,T478,N481,V483,E484,G485,F486,N487,Y489,F490,L492,Q493,S494,Q498,T500

A123,G142,Y144,F157,R158,N164

F140,Y144

K417,E484,N501,R683

N440

E484,F490,Q493

E484,Q493

R346,N440

R403,D405,T415,G416,K417,D420,Y421,Y453,L455,F456,R457,K458,N460,Y473,A475,G476,F486,N487,Y489,Q493,S494,Y495,G496,Q498,T500,N501,G502,G504,Y505

E484

F377

F377,E516

F377,K386,L390

F377,T385,L390

F377,Y508,E516

F486

F490

G446

G476,N501,Y508

K444

L441

L452

N354,F377,I468

N354,K356,I468

N450

P499

R403,K417,Y449,N450,L452,Y453,L455,F456,E484,G485,F486,Y489,F490,L492,Q493,S494,Y495,Y505

S477

S477,S514

S494

T345

T478

V341,F377,N501,V503,Y508,E516

V503

R403,R408,T415,G416,K417,D420,Y421,Y453,L455,F456,R457,K458,N460,Y473,Q474,A475,G476,F486,N487,Y489,Q493,S494,Y495,G496,Q498,T500,N501,G502,Y505

R403,T415,G416,K417,D420,Y421,Y453,L455,F456,R457,K458,N460,Y473,A475,G476,F486,N487,Y489,Q493,S494,Y495,G496,Q498,T500,N501,G502,Y505

R403,T415,G416,K417,D420,Y421,Y453,L455,F456,R457,K458,N460,Y473,A475,G476,S477,F486,N487,Y489,Q493,Q498,T500,N501,G502,Y505

R403,T415,G416,K417,D420,Y421,Y453,L455,F456,R457,K458,N460,Y473,Q474,A475,G476,F486,N487,Y489,Q493,S494,Y495,G496,Q498,T500,N501,G502,Y505

E484

F456

F490

G446

G485

P384

R403,D405,E406,R408,Q409,T415,G416,K417,D420,Y421,L455,F456,R457,K458,N460,Y473,Q474,A475,G476,S477,F486,N487,Y489,Q493,Y495,G502,Y505

S494

C136,N137,D138,P139,F140,L141,G142,V143,T307,V308,E309,K310,G311,I312,Y313,Q314,T315,S316,N317,F318,P621,V622,A623,I624,H625,A626,D627,Q628,L629,T630,P631,T632,W633,R634,V635,Y636

C136,N137,D138,P139,F140,L141,G142,V143,Y144,L242,L244,H245,R246,S247,Y248,L249,T250,P251,G252,D253,S254,S255,S256,G257,W258,T259,A260,G261,A262,A263,A264,Y265

C136,N137,D138,P139,F140,L141,G142,V143,Y144,V171,S172,Q173,P174,F175,L176,M177,D178,L179,L242,A243,L244,H245,R246,S247,Y248,L249,T250,P251,G252,D253,S254,S255,S256,G257,W258,T259,A260,G261,A262,A263,A264

R403,D405,T415,G416,K417,D420,Y421,L455,F456,R457,K458,S459,N460,Y473,Q474,A475,G476,S477,F486,N487,Y489,N501,G502,Y505

R403,T415,G416,K417,D420,Y421,Y453,L455,R457,K458,N460,Y473,A475,G476,S477,F486,N487,Y489,Q493,S494,Y495,G496,Q498,T500,N501,G502,V503,Y505

V433,I434,A435,W436,N437,S438,N439,N440,L441,D442,S443,K444,V445,G446,G447,N448,Y449,N450,Y451,L452,Y453,R454,L455,G496,F497,Q498,P499,T500,N501,G502,V503,G504,Y505,Q506,P507,Y508,R509,V510,V511,V512,L513

Y351,Y449,L455,T470,N481,G482,V483,E484,G485,F486,C488,Y489,F490,L492,Q493,S494

Y369,S371,F377,K378,C379,Y380,G381,V382,S383,P384,T385,R408,P412,G413,Q414,T415,G416,D427,D428,F429

T345,N439,N440,S443,K444,V445,G446,G447,N450,Q498,P499,T500,Q506

Y351,K444,V445,G446,G447,N448,Y449,N450,L452,T470,E484,F490,L492,Q493,S494,Q498

D405,K417,D420,L455,F456,N460,I472,Y473,A475,G476,F486,N487,Y489,G504

G446,G447,Y449,F456,T470,V483,E484,G485,F486,C488,Y489,F490,P491,L492,Q493,S494,Q498

K417

K417,L455,F456,T470,E471,I472

K444,V445,G446

K444,V445,G446,L452,L455,F456,T470,E471,I472,S494

L455,F456,S494,N501

L455,F456,T470,E471,I472

N501

T470,E471,I472

T470,E471,I472,N501

K147,E484,N501

N343,A344,T345,R346,S373,W436,N437,N440,L441,S443,K444,V445,N448,N450,R509

Y369,N370,S371,F377,K378,C379,Y380,G381,V382,S383,P384,T385,R408,P412,G413,Q414,T415,G416,D427,D428,F429

F140,G142,V143,Y145,H146,N148,N149,W152,E154,F157,A243,L244,H245

S12,C15,L18,T19,C136,G142,H146,K147,N149,R246

V16,N17,F140,G142,V143,Y144,Y145,H146,K147,N148,W152,E154,E156,R158,L244,H245,R246,L249,P251

V16,N17,T19,Y144,R246,S247,Y248,T250,P251,G252,D253,S254,S255,S256,G257

V16,N17,T20,Y144,Y145,H146,K147,N148,S155,R158,R246,L249,T250,P251,G252,D253

Y351,G446,Y449,N450,L452,F456,T470,T478,P479,C480,N481,G482,V483,E484,G485,F486,N487,C488,Y489,F490,P491,L492,Q493,S494

Y369,N370,S371,A372,F374,S375,T376,F377,K378,C379,S383,P384,D405,R408,Q409,Q414,T415,G416,N501,V503,G504,Y505

Y449,L452,T470,E471,I472,N481,G482,V483,E484,G485,F486,F490,L492,Q493,S494

K417,E484,N501

L452,L455,F456,I472,N481,G482,V483,E484,G485,F486,Y489,F490

L455,F456,K458,Y473,A475,G476,S477,T478,G485,F486,N487,Y489,Q493

L455,K458,Y473,A475,G476,S477,T478,G485,F486,N487,C488,Y489,Q493

N334,L335,P337,G339,E340,N343,A344,T345,K356,R357,S359,C361,N440,L441

Q14,Y144,Y145,H146,K147,F157,G252,D253

R403,D405,E406,R408,Q409,T415,G416,K417,D420,Y421,Y453,L455,F456,R457,K458,N460,Y473,Q474,A475,G476,S477,F486,N487,Y489,Y495,G496,Q498,T500,N501,G502,V503,Y505

R403,D405,R408,T415,G416,K417,D420,Y421,Y453,L455,R457,K458,N460,Y473,Q474,A475,G476,S477,F486,N487,Y489,Q493,Y495,G496,Q498,T500,N501,G502,Y505

R403,E406,Q409,T415,G416,K417,D420,Y421,Y453,L455,F456,R457,K458,N460,Y473,Q474,A475,G476,F486,N487,Y489,Q493,S494,Y495,G496,Q498,T500,N501,G502,Y505

R403,K417,Y453,L455,F456,E484,G485,F486,N487,C488,Y489,Q493,N501,G502,Y505

R403,T415,G416,K417,D420,Y421,Y453,L455,F456,R457,K458,S459,N460,Y473,Q474,A475,G476,S477,F486,N487,Y489,Y495,G496,Q498,T500,N501,G502,Y505

W353,N354,R355,K356,R357,S359,N360,N394,Y396,P426,D428,K462,P463,F464,E465,R466,I468,E516,L518,H519,A520,T523

Y449,L455,F456,V483,E484,G485,F486,Y489,F490,L492,Q493,S494

Y473,A475,T478,F486,N487

D614

G446,N448,Y449,L452,E484,G485,F486,N487,Y489,F490,L492,Q493,S494

R403,D405,E406,R408,T415,G416,K417,D420,Y421,Y449,L455,F456,R457,K458,N460,Y473,Q474,A475,G476,S477,N487,Y489,Q493,S494,G496,T500,N501,G502,V503,Y505

R403,D405,R408,Q409,T415,G416,K417,Y421,Y449,Y453,L455,F456,G485,F486,N487,Y489,Q493,S494,Y495,G496,N501,Y505

R403,E406,R408,Q409,G416,K417,Y449,Y453,L455,F456,F486,N487,Y489,Q493,S494,Y495,G496,Q498,T500,N501,G502,Y505

R403,K417,Y449,L452,Y453,L455,F456,E484,G485,F486,C488,Y489,F490,L492,Q493,Y505

R403,R408,Q409,Q414,T415,G416,K417,D420,Y421,G446,Y449,F456,A475,G476,S477,F486,N487,Y489,Q493,S494,G496,Q498,N501,Y505

R403,T415,G416,K417,D420,Y421,L455,F456,R457,N460,Y473,A475,G476,F486,N487,Y489,Q493,G496,T500,N501,G502,Y505

T415,G416,K417,D420,Y421,L455,F456,R457,K458,N460,Y473,Q474,A475,G476,S477,F486,N487,G496,Y505

V483,E484,F486,Y489

Y144

Y421,F456,R457,Y473,A475,G476,S477,E484,G485,F486,N487,Y489,Q493

K417,E484

K417,N501

L18,D80,L242,A243,L244,R246

L18,Y144,L242,A243,L244,N501

L18,Y144,L242,A243,L244,R246,E484,N501,A701,T716

L242,A243,L244,E484

S982

Y144,L242,A243,L244,R246

Y144,L242,A243,L244,R246,A570

Q14,C15,V16,N17,T19,G142,V143,Y144,K147,E156,R158,L244,H245,R246,S247,Y248,L249,T250,P251,G252,S256

Q14,C15,Y144,H146,K147,E154,E156,R158,R246,Y248,L249,T250,P251,D253,S254

Q14,N17,L18,T76,K77,V143

Q14,Y144,H146,K147,N148,N149,W152,M153,E154,E156,F157,R158,R246,Y248,L249,P251,G252,D253

Y144,Y145,H146,K147,K150,W152,H245,R246,Y248,L249,T250,P251,G252,S254,S255,S256

Y144,Y145,H146,K147,N148,K150,R246,S247,Y248,L249,T250,P251,G252,D253,S254

G446,Y449,L452,T478,V483,E484,G485,F486,N487,Y489,F490,L492,Q493,S494,G496,Q498

R403,D405,R408,T415,G416,K417,D420,Y421,L455,F456,R457,K458,N460,Y473,Q474,A475,G476,S477,F486,N487,Y489,Q493,T500,N501,G502,Y505

W353,R355,R357,Y396,P426,D427,D428,F429,K462,P463,F464,R466,S514,E516,L518,H519,A520,P521

Y144,W152,R246,Y248

Y144,Y145,H146,K147,R246,S247,Y248,L249,T250,P251,G252,S255

Y145,K147,W152,Y248

Y369,N370,S371,A372,F374,S375,T376,F377,K378,C379,Y380,V382,S383,P384,T385,G404,D405,R408,T500,N501,G502,V503,G504,Q506

Y449,L455,F456,V483,E484,G485,F486,Y489,F490,Q493,S494

K444,G447

N487

K304,S371,N388

K417,E484,N501

N440

R403,D405,A419,G502,G504,Y505,R509

S71,K97,S98,T124,Y145,H146,K147,K150,S151,W152,E180,G181,K182,Q183,N185,V213,H245,S247,Y248,L249,T259,A260,A262

F374,S375,T376,F377,K378,C379,Y380,G381,V382,S383,P384,T385,K386,R408,N437,V503,G504,Y508

R346,Y351,K444,Y449,N450,L452,T470,I472,N481,G482,V483,E484,F490,L492,S494

R403,T415,G416,K417,D420,Y421,Y453,L455,F456,R457,K458,N460,Y473,Q474,A475,G476,S477,F486,N487,Y489,Q493,S494,Y495,G496,Q498,T500,N501,G502,Y505

S477

N439,N440,S443,K444,V445,G446,G447,Y449,N450,S494,P499,T500,Q506

R403,T415,G416,K417,D420,Y421,Y453,L455,F456,R457,K458,N460,Y473,Q474,A475,G476,S477,F486,N487,Y489,Q493,Y505

T415,Y421,A475,G476,N487,S494,G502

Y369,N370,F374,S375,T376,F377,K378,C379,Y380,G381,V382,S383,P384,T385,K386,F429,T430,F515,L517

E484,N501

K417

K417,E484,N501

R346,N439,N440,S443,K444,V445,G446,G447,N450,Q498,P499,T500,N501,G502,Q506

D405,K417,L455,F456,A475,G476,S477,E484,G485,F486,N487,Y489

F456,Y473,A475,G476,S477,T478,V483,E484,G485,F486,N487,C488,Y489

K417,N501

L242,A243,L244

N501

R346,K444,Y449,N450,L452,I472,N481,G482,V483,E484,F490,L492

R403,D405,T415,G416,K417,D420,Y421,L455,F456,R457,K458,N460,Y473,A475,G476,S477,E484,F486,N487,Y489,F490,Q493,S494,G496,Q498,N501,G502,Y505

R403,D405,T415,G416,K417,D420,Y421,Y453,L455,F456,R457,K458,S459,N460,Y473,A475,G476,S477,F486,N487,Y489,Q493,G496,Q498,T500,N501,G502,Y505

R403,T415,K417,D420,Y421,L455,R457,K458,N460,Y473,Q474,A475,G476,S477,T478,F486,N487,Y489,N501,G502,Y505

E484

N501

A372,K378

Q493

K417,L455,F456,Y473,Q474,A475,G476,S477,T478,C480,E484,G485,F486,C488,Y489,Q493

K444,G446,S494,N450

L455,F456,A475,G476,S477,T478,E484,F486,N487,Y489

E340,V341,N343,T345,R346,F347,A348,Y351,N354,K356,L441,Y449,N450,R466,I468,T470,N481,G482,V483,E484,F490

Q14,Y144,Y145,H146,K147,M153,F157,R158,R246,Y248,L249,T250,P251,G252,D253,S254

Y351,K444,G446,Y449,N450,L452,T470,V483,E484,G485,F486,N487,C488,Y489,F490,L492,Q493,S494,Q498

Y369,A372,S373,F374,S375,T376,F377,K378,C379,P384,R408,N437,G502,V503,Q506,Y508

I472,E484,F486,N487,Y489,F490

K417,L455,F456,Y473,I472,A475,E484,F486,N487,Y489,Q493

K417,Y449,L455,F456,Y489,G496,Q498,T500,N501

L455,Y473,A475,G476,S477,T478,E484,G485,F486,N487,C488,Y489,Q493

N331,I332,T333,N334,L335,P337,G339,E340,N343,A344,T345,R346,K356,R357,S359,C361,L441

S443,V445,G446,P499

Y351,Y449,L452,F456,T470,G482,E484,G485,F486,Y489,F490,L492,S494

Y369,S371,A372,F374,S375,T376,F377,K378,C379,V382,S383,P384,R403,G404,D405,R408,N501,G502,V503,G504,Y505,Y508

Y369,S375,F377,K378,C379,Y380,G381,V382,S383,P384,T385,K386,L390,C391,F392,P412,D427,D428,F429,L517,K528

Y449,F456,I472,Y473,N481,V483,E484,G485,F486,F490

Y449,N450,L452,E484,F490,S494

D614

E484

F140

F140,E484

S13,W152

E484,S494,N501

A475

A831

D614,I472

F490

H519

L452

N234

N439

V483

E337,T342,R343,F344,A345,Y348,N351,K353,Y446,N447,L449,R463,I465,T467,V480,F487

E337,T342,R343,Y348,N351,R352,K353,R354,Y446,N447,L449,R463,I465,S466,T467,E468,G479,F487,L489

L452,F453,Y470,A472,G473,S474,T475,V480,G482,F483,N484,C485,Y486,Q490

R343,N436,N437,L438,D439,S440,K441,V442,G443,G444,P496,T497

R400,D402,E403,R405,Q406,T412,G413,N414,D417,Y418,L452,F453,R454,K455,N457,Y470,A472,G473,S474,T475,F483,N484,Y486,Q490,Y498,Y502

T342,R343,N436,N437,L438,K441,V442,G443,G444,N447,Q495,P496,T497

T342,R343,Y348,L438,K441,V442,G444,Y446,N447,L449,I465,T467,G479,K481,F487,L489,S491

T412,G413,N414,D417,Y418,Y446,L452,F453,T475,P476,K481,G482,F483,N484,Y486,Q490,S491

G339,E340,N343,A344,T345,R346,N437,N440,L441,D442,S443,K444,V445,N448,Y451,P499,T500

G485,F486

R346,K444,G446

R403,T415,G416,K417,D420,Y421,L455,F456,R457,K458,N460,Y473,A475,G476,S477,F486,N487,Y489,Q493,Y495,T500,N501,G502,V503,Y505

R403,T415,G416,K417,D420,Y421,Y453,L455,F456,R457,K458,N460,Y473,A475,G476,S477,F486,N487,Y489,Q493,T500,N501,G502,G504,Y505

Y369,S371,A372,F374,S375,F377,K378,C379,Y380,G381,V382,S383,P384,T385,V407,R408,P412,G413,D427,D428,F429,T430,V503,G504

Y369,S371,F377,K378,C379,Y380,G381,V382,S383,P384,T385,R408,P412,G413,Q414,T415,G416,D427,D428,F429,T430

F342,T345,R346,L368,S373,F374,W436,N437,N440,L441,K444,V445

T345,R346,N439,N440,L441,S443,K444,V445,G446,G447,N450,Q498,P499,T500,Q506

Y369,S371,A372,F377,K378,C379,Y380,G381,V382,S383,P384,R408,P412,G413,Q414,T415,D420,D427,N460

A348,N450,L452

F456,N487

G476,T478,G485,F486,N487

K417,E484,N487,N501

K417,F456,N460,A475,N487

K417,F456,N487

K417,L452,F490,N501

L452,E484

N343,T345,R346,W436

R346,N450,L452,E484,F490

R403,R408,K417,N501,Y505

Y449,L452,F490

E484,F486

E484,N501

H69,V70,Y144,L242,A243,L244,G261

K417

K417,E484

K417,E484,F486

K417,N439,Y453,E484

K417,N501

K417,Y453,S477,E484,F486

L242,A243,L244

N439,E484,N501

N439,Y453,E484

N439,Y453,T478,E484,N501

S477,T478,F486

V367,N439

V367,N439,Y453

W353,N354,R355,R357,Y449,N450,L452,R457,P463,F464,E465,R466,D467,I468,S469,T470,I472,G482,E484,F490,L492

Y144,L242,A243,L244

Y144,L242,A243,L244,G261

Y144,Y145,H146,K147,K150,W152,R246,S247,Y248,L249

K417,Y421,L455,F456,R457,K458,Y473,Q474,A475,G476,T478,P479,C480,V483,E484,G485,F486,N487,C488,Y489,F490,Q493

Y145,K150,W152

Y351,A352,W353,N360,L368,A419,V433,Y449,N450,D467,C480,E484,C488,F490,S494,R509

N354,R355,R357,D428,L461,K462,P463,F464,E465,R466

E406

F486

Q493

L455,F456,A475,G476,S477,T478,P479,E484,G485,F486,N487,Y489,Q493

E484,G485

K417,Y453,L455,F456,Y473,A475,T478,E484,G485,F486,N487,Y489,Q493

K444,G446,Y449,N450,L452,V483,E484,G485,F486,Y489,F490,L492,Q493,S494,Y495,G496,Q498,Y505

Q14,V16,Y144,K147,R246,Y248,P251,G252,D253

R355,R457,S459,K462,P463,F464,E465,R466,D467,I468,S469,E471,Q474,P479,C480,N481,G482,L518

R403,K417,Y449,N501,Y505

T415,G416,K417,D420,Y421,L455,F456,R457,K458,N460,Y473,Q474,A475,G476,S477,F486,N487,Y489,Q493,Y505

W353,R355,Y396,R457,K462,P463,F464,E465,R466,D467,I468,E516,L518

Y144,Y145,H146,K147,K150,W152,Y248

Y145,H146,K147,R246,Y248,P251,G261

Y145,K147,W152

Y145,K147,Y248

Y369,N370,S371,A372,F374,S375,T376,F377,K378,C379,P384,T385,N388

K417,Y449,L452,L455,F456,E484,G485,F486,N487,C488,Y489,F490,L492,Q493,S494,G496,Q498,N501,G502,Y505

A152,S154,T155,P156,C157,V160,K161,G162,F163,N164,C165,Y166

F133,K161,G162,F163,Y166,Q170

G123,Y126,Q170,S171,Y172,Q175,P176,T177,Y178,G179,Y182

G16,E17,N20,A21,T22,R23,S50,F51,W113,N117,L118,D119,S120,K121,V122,N125,N127,Y128

N117,S120,K121,V122,G123,Q175,P176,T177,Y178,G179,Y182

R80,D82,R85,D97,Y98,Y130,L132,F133,R134,Y150,F163,N164,Y166,Q170,S171,Y172,G173,Y178,G179,V180,Y182

R80,R85,T92,D97,Y98,L132,F133,F163,N164,Y166,F167,Q170,S171,Y172,G173,T177,Y178,G179,Y182

R80,T92,G93,N94,D97,Y98,L132,F133,R134,K135,S136,N137,Y150,Q151,A152,G153,S154,F163,N164,Y166,Q170,T177,Y178,G179,Y182

S52,R80,G81,D82,R85,V180,G181,Y182,Y185

T10,N11,L12,C13,P14,F15,G16,E17,F19,N20,T22,R23,V39,A40,D41,Y42,V44,L45,K205

T10,N11,L12,C13,P14,F15,G16,E17,F19,N20,T22,V39,A40,D41,Y42,V44,L45

T92,Y98,L132,F133,R134,K135,Y150,A152,G153,S154,T155,P156,V160,G162,F163,N164,Q170

V122,G123,Y126,G173,Q175,P176,T177,Y178

V122,G123,Y126,Q170,Q175,P176,T177,Y178,G179,Y182

Y28,Y126,N127,L129,T147,I149,P156,C157,N158,G159,V160,K161,F163,F167,L169

S13,Q14,C15,V16,N17,F18,T19,T76,F140,Y144,Y145,H146,K147,N148,M153,S155,E156,R158,L246,T247,P248

A344,T345,R346,F347,A348,S349,Y351,A352,N440,K444,V445,G446,G447,N448,Y449,N450

G485,F486,N487

R403,T415,G416,D420,Y421,L455,F456,R457,K458,N460,Y473,Q474,A475,G476,S477,E484,F486,N487,Y489,F490,L492,Q493,N501,G502,Y505

T345,R346,N440,L441,S443,K444,V445,N448,N450,Y451,P499,T500

A123,T124,Y144,Y145,H146,K147,K150,S151,W152,M153

N331,I332,T333,N334,L335,P337,G339,E340,N343,A344,T345,R346,K356,R357,S359,C361,L441

Y351,Y449,L455,T470,N481,G482,V483,E484,G485,F486,C488,Y489,F490,L492,Q493,S494

Y369,N370,S371,A372,S373,F374,S375,T376,F377,K378,C379,S383,P384,T385,N437

L455,K458,A475,G476,S477,T478,V483,E484,F486,N487,Q493

L455,K458,Y473,A475,G476,S477,T478,V483,E484,G485,F486,N487,Q493

N343,T345,R346,N439,N440,L441,D442,S443,K444,V445,N448,Y451,P499,T500,R509

N439,N440,L441,D442,S443,K444,V445,G446,G447,N448,Y449,N450,Y451,L452,Y453,R454,L455,F456,R457,K458,P499,T500,N501,G502,V503,G504,Y505,Q506,P507,Y508,R509,V510,V511,V512,L513,S514,F515,E516,L517,L518

N99,R102,G103,W104,I119,N121,Y170,S172,Q173,P174,F175,L176,M177,D178,E180,K182,N188,R190,F192,H207,E224,L226

R346,N439,N440,L441,S443,K444,V445,G446,G447,N448,Y449,N450,Q498,P499,T500

Y369,N370,S371,A372,F374,F377,K378,C379,Y380,G381,V382,S383,P384,T385,K386,L390,F429,T430,F515,E516,L517

F456,A475,G476,S477,T478,E484,G485,F486,N487,Y489

E484,F490

E484,Q493

G446

G476,F486

K417,Q493

K444

K444,G446

K444,G446,L452

L452,E484

L452,E484,F490

L452,Q493

R403,Q409,T415,G416,K417,D420,Y421,Y453,L455,F456,R457,K458,N460,Q474,F486,N487,Y489,Q493,S494,G496,N501,V503,Y505

G443

K441

K481

L449

N414

Y498

L455,A475,G476,S477,T478,E484,G485,F486,N487,C488,Y489,Q493

R346,N439,N440,L441,S443,K444,V445,G446,Y449,N450,L452,E484,F490,Q493,S494,P499

R403,D405,E406,R408,T415,G416,K417,D420,Y421,Y449,Y453,L455,F456,R457,K458,N460,Y473,A475,G476,S477,F486,N487,Y489,Q493,S494,Y495,G496,Q498,T500,N501,G502,Y505

R403,D405,T415,G416,K417,D420,Y421,Y453,L455,F456,R457,K458,N460,Y473,Q474,A475,G476,S477,F486,N487,Y489,Q493,G502,Y505

R403,T415,G416,K417,D420,Y421,Y453,L455,F456,R457,K458,N460,Y473,A475,G476,S477,F486,N487,Y489,Q493,Y495,G496,Q498,T500,N501,G502,V503,Y505

D402,R405,T412,G413,N414,D417,Y418,L452,F453,R454,K455,N457,Y470,A472,G473,F483,N484,Y486,Q490,Y498,G499,Y502

L452,Y470,A472,G473,S474,T475,G482,F483,N484,C485,Y486,Q490

I332,T333,N334,L335,C336,P337,G339,E340,V341,N343,A344,T345,R346,N354,K356,R357,I358,S359,N360,C361,N440,L441,R509

K417,Y421,Y449,L452,L455,F456,A475,G476,T478,E484,G485,F486,N487,C488,Y489,F490,L492,Q493,S494,G496,Q498,N501,G502,Y505

L455,F456,Q474,A475,G476,S477,T478,P479,E484,G485,F486,N487,C488,Y489,Q493

R403,D405,E406,Q409,T415,G416,K417,D420,Y421,Y453,L455,F456,R457,K458,N460,Y473,A475,G476,S477,F486,N487,Y489,Q493,S494,Y495,G496,Q498,T500,N501,G502,V503,Y505

R403,D405,E406,R408,Q409,T415,G416,K417,D420,Y421,Y453,L455,F456,R457,K458,S459,N460,Y473,Q474,A475,G476,S477,F486,N487,Y489,Q493,S494,Y495,T500,N501,G502,V503,G504,Y505

R403,K417,G446,Y449,N450,L452,Y453,L455,F456,V483,E484,G485,F486,Y489,F490,L492,Q493,S494,Y495,Y505

T345,R346,N439,N440,L441,S443,K444,V445,G446,G447,Y449,N450,L452,E484,F490,L492,Q493,S494,P499

W353,R355,T393,N394,Y396,P426,D427,D428,F429,T430,K462,P463,F464,S514,F515,E516,L518,H519,A520,P521

Y351,Y449,N450,L452,L455,F456,R457,T470,I472,N481,G482,V483,E484,G485,F486,N487,C488,Y489,F490,L492,Q493,S494,Y495,G496,Y369,N370,S371,A372,S373,F374,S375,T376,F377,K378,C379,Y380,V382,S383,P384,T385,K386,L387,G404,D405,R408,Q409,G502,V503,G504,Q506,Y508

L242,A243,L244

R403,D405,E406,R408,T415,G416,K417,D420,Y421,Y449,Y453,L455,F456,R457,K458,N460,Y473,A475,G476,S477,F486,N487,Y489,Q493,S494,Y495,G496,Q498,T500,N501,G502,Y505

R403,D405,T415,G416,K417,D420,Y421,Y453,L455,F456,R457,K458,N460,Y473,Q474,A475,G476,S477,F486,N487,Y489,Q493,G502,Y505

R403,T415,G416,K417,D420,Y421,Y453,L455,F456,R457,K458,N460,Y473,A475,G476,S477,F486,N487,Y489,Q493,Y495,G496,Q498,T500,N501,G502,V503,Y505

R400,T412,G413,N414,D417,Y418,Y450,L452,F453,R454,K455,N457,Y470,Q471,A472,G473,S474,F483,N484,Y486,Q490,T497,Y498,G499,Y502

R403,D405,R408,T415,G416,K417,D420,Y421,Y453,L455,F456,R457,K458,N460,Y473,Q474,A475,G476,S477,F486,N487,Y489,Q493,N501,G502,Y505

E340,A344,T345,R346,F347,A348,Y351,A352,N354,K356,Y449,N450,R466,I468,T470,N481,F490

T345,R346,F347,A348,S349,Y351,N440,L441,D442,S443,K444,V445,N448,N450,Y451,R509

L452,L455,F456,I472,N481,G482,V483,E484,G485,F486,Y489,F490

N331,I332,T333,N334,L335,P337,G339,E340,N343,A344,T345,R346,K356,R357,S359,C361,L441

Q14,Y144,Y145,H146,K147,F157,G252,D253

R403,D405,E406,R408,Q409,T415,G416,K417,D420,Y421,Y453,L455,F456,R457,K458,N460,Y473,Q474,A475,G476,S477,F486,N487,Y489,Y495,G496,Q498,T500,N501,G502,V503,Y505

R403,D405,R408,T415,G416,K417,D420,Y421,Y453,L455,R457,K458,N460,Y473,Q474,A475,G476,S477,F486,N487,Y489,Q493,Y495,G496,Q498,T500,N501,G502,Y505

R403,E406,Q409,T415,G416,K417,D420,Y421,Y453,L455,F456,R457,K458,N460,Y473,Q474,A475,G476,F486,N487,Y489,Q493,S494,Y495,G496,Q498,T500,N501,G502,Y505

R403,K417,Y453,L455,F456,E484,G485,F486,N487,C488,Y489,Q493,N501,G502,Y505

R403,T415,G416,K417,D420,Y421,Y453,L455,F456,R457,K458,S459,N460,Y473,Q474,A475,G476,S477,F486,N487,Y489,Y495,G496,Q498,T500,N501,G502,Y505

Y351,Y449,L455,T470,N481,G482,V483,E484,G485,F486,C488,Y489,F490,L492,Q493,S494

Y449,L455,F456,V483,E484,G485,F486,Y489,F490,L492,Q493,S494

R403,D405,R408,T415,G416,K417,D420,Y421,L455,F456,R457,K458,N460,Y473,Q474,A475,G476,S477,F486,N487,Y489,Q493,T500,N501,G502,Y505

T345,R346,Y351,N440,L441,D442,S443,K444,V445,G446,G447,N448,Y449,N450,L452,T470,F490,P499

Y453,L455,Y473,A475,G476,S477,T478,G485,F486,N487,Y489,Q493

L455,A475,G476,S477,T478,E484,G485,F486,N487,C488,Y489,Q493

R346,N439,N440,L441,S443,K444,V445,G446,Y449,N450,L452,E484,F490,Q493,S494,P499

R403,D405,R408,Q498,T500,N501,G502,V503,G504,Y505,Q506

Y369,N370,S371,A372,F374,S375,T376,F377,K378,C379,S383,P384,D405,R408,Q409,Q414,T415,G416,N501,V503,G504,Y505

Y369,N370,S371,A372,F374,S375,T376,F377,K378,C379,Y380,V382,S383,P384,T385,G404,D405,R408,T500,N501,G502,V503,G504,Q506

D405,K417,D420,L455,F456,N460,I472,Y473,A475,G476,F486,N487,Y489,G504

K417,L455,F456,Y473,A475,S477,T478,E484,G485,F486,N487,Y489,F490,L492,Q493

Y369,N370,A372,F374,S375,T376,K378,C379,G404,D405,V407,R408,G502,V503,G504,Y508

K417,L455,F456,Y473,Q474,A475,G476,S477,T478,G485,F486,N487,C488,Y489,Q493

T345,R346,N439,N440,L441,D442,S443,K444,V445,N448,Y451,P499,T500

T342,R343,N436,K437,L438,D439,S440,K441,V442,N445,N447,Y448,P496,T497

F43,F175,L176,L226

R102,Y145,K147,W152,R246,Y248,P251,G252

Y366,A369,P370,F371,F372,T373,K375,Y377,S380,P381,T382,K383,G401,D402,V404,R405,I407,A408,V430,A432,V500,G501,Y505,V507

> LINEAR EPITOPES

PSKPSKRSFIEDLLFNKV

DISTEIYQAGSTPCNGVEGFNCYFPLQSYGFQPTNGVGYQPYRVVVL

IYQAGSTPCNGVEGFNCYFPLQSY

DSFKEELDKYFKNHTS

PLQPELDSFKEELDKYFKNHTSPDV

KEELDKYFKNHTSPDVD

TEIYQAGSTPCNGVEGF

KRSFIEDLLFNK

SFKEELDKYF

DKYFKNHTSPDVDL

PPLLTDEMIAQYTSA
