# Supplementary material for: The fourth COVID-19 vaccine dose increased the neutralizing antibody response against the SARS-CoV-2 Omicron (B.1.1.529) variant in a diverse Brazilian population
Source: Microbiol Spectr. 2023 Nov 1;11(6):e02857-23. doi: 10.1128/spectrum.02857-23 (PMC10714775; doi:10.1128/spectrum.02857-23)
Supplement: Supplementary Figure 1 legend — Legend for Supplementary Figure 1. [file spectrum.02857-23-s0006.docx]

**Supplementary figure 1- Serological analyses according to the time of sample collection following the last vaccine dose.** Serum levels of SARS-CoV-2-specific antibodies measured by ELISA in samples from volunteers vaccinated with three or four doses, respectively (A and B). Neutralizing antibody titers measured in serum samples from volunteers vaccinated with three or four doses, respectively (C and D). Medians of groups were compared using Kruskal–Wallis test followed by Dunn's multiple comparisons (C and D). Statistical significance was set at p ≤ 0.05. Statistical power was set to be of at least 80%. Standard deviations (SD) are given as error bars.
